# Factors Associated with Anchor-Number Preference Among Orthopedic Surgeons Performing Bankart Repair in Saudi Arabia: A Cross-Sectional Survey

**DOI:** 10.3390/healthcare14193304

**Published:** 2026-10-03

**Authors:** Ismail Almogbil, Lama AlOraini, Abdulhakeem AlKharraz, Shaden Alenezi, Nejood AlFehaid, Yumna Almishary, Raghad Al-Awn, Abdullah AlShahrani

**Affiliations:** 1 Department of Orthopedic Surgery, College of Medicine, Qassim University, Buraydah 52571, Saudi Arabia; 2 Department of Orthopedic Surgery, King Fahad Specialist Hospital, Buraydah 52366, Saudi Arabia; 3 College of Medicine, Qassim University, Buraydah 52571, Saudi Arabia; 4 College of Medicine, University of Ha’il, Ha’il 55476, Saudi Arabia; 5 College of Medicine, Sulaiman AlRajhi University, Al Bukayriah 51941, Saudi Arabia; 6 Department of Orthopedic Surgery, Armed Forces Hospital, Khamis Mushait 62413, Saudi Arabia

**Keywords:** shoulder dislocation, arthroscopy, suture anchors, orthopedic surgeons, cross-sectional studies

## Abstract

**Objective:** This study described anchor-number preferences among orthopedic surgeons performing arthroscopic Bankart repair in Saudi Arabia and explored unadjusted associations with surgeon- and practice-related characteristics. **Methods:** A cross-sectional electronic survey used purposive recruitment across five Saudi regions. Descriptive analyses included 71 respondents. Because ‘Both’ represented case-dependent use rather than a discrete anchor count, these three responses were reported descriptively and excluded from inferential comparisons; exact analyses compared 1–2 anchors (n = 18) with 3–4 anchors (n = 50). Fisher–Freeman–Halton exact tests, Cramér’s V, and bootstrap 95% confidence intervals were reported. **Results:** Fifty surgeons (70.4%; 95% CI: 59.0–79.8%) reported usually using 3–4 anchors. In exploratory unadjusted analyses, anchor-number preference was associated with annual Bankart-repair volume (exact *p* = 0.021; Cramér’s V = 0.371, 95% CI: 0.194–0.548) and hypothetical future preference when cost and other factors were not considered (exact *p* = 0.044; Cramér’s V = 0.298, 95% CI: 0.068–0.529). Other assessed associations were nonsignificant. Tear size and extent was the most frequently reported consideration (95.8%). **Conclusions:** Most respondents preferred 3–4 anchors, and tear size/extent was the most frequently reported consideration. The observed associations were exploratory and unadjusted and did not establish independent determinants, cost effects on actual practice, superior stabilization, or guideline recommendations.

## 1. Introduction

The shoulder joint is the most frequently dislocated joints in the human body. The most common type of shoulder dislocation is anterior dislocation. A Bankart lesion (BL) causes anterior shoulder dislocation in 87 to 100% of cases [1]. BL is characterized by anteroinferior labrum detachment. There are two types: bone BL and soft tissue BL. A bone BL is defined as an anteroinferior glenoid rim fracture, whereas a soft tissue BL is defined as an avulsion injury to the glenoid rim [2].

Currently, arthroscopic Bankart repair is the most popular surgical method for addressing symptomatic shoulder instability [3,4,5]. According to the American Board of Orthopedic Surgery (ABOS), there has been a considerable increase in arthroscopic Bankart repairs when compared to open surgeries [3]. Arthroscopic stabilization of BL with suture anchors is the preferred treatment for primary anterior glenohumeral instability by many surgeons due to the 9% mid- to long-term recurrence rate, low risk of comorbidities and lower morbidity. In comparison with open surgeries, arthroscopic stabilization is associated with less iatrogenic damage, less postoperative pain, better cosmesis, and lower morbidity in selected patients [4,5].

If there is no bone deficit, the sutures are anchored to repair the labrum in arthroscopic surgery [6]. Sutures are used as an effective means to secure the soft tissues to bones [7]. It is increasingly being recognized among surgeons that there is a need to determine the perfect number of suture anchors for shoulder stabilization [8]. However, this depends on the skill and expertise of the surgeon [6].

Various published reports have described acceptable results using fewer than three anchors in selected cases [8,9,10,11,12], but some have suggested using three sutures [6,9], while some have recommended using four anchors in some specific cases [10]. Such controversies arise due to differences in the extent of tear, glenoid bone defect, patient risk factors, capsular pathology, anchor configuration, surgical technique and criteria used for outcome assessment.

There is no consensus regarding the number of anchors for arthroscopic Bankart repair, and practice patterns in Saudi Arabia remain incompletely described. This study therefore described surgeons’ usual anchor-number preferences and explored unadjusted associations between the mutually exclusive 1–2- and 3–4-anchor categories and surgeon-, practice-, and case-related characteristics. Case-dependent ‘Both’ responses were retained descriptively but were not treated as a third count category in inferential analyses. We hypothesized that 3–4 anchors would be the most frequently reported preference and that preference would vary across selected practice characteristics; no causal or independent-factor inference was intended.

## 2. Materials and Methods

### 2.1. Study Design and Setting

This was a cross-sectional study conducted across five major regions of the Kingdom of Saudi Arabia: Central, Eastern, Western, Southern, and Northern regions. The study encompassed a diverse range of healthcare institutions, including government hospitals, private clinics, and specialized orthopedic centers, ensuring a broad representation of orthopedic practices. The study was conducted from 6 December 2024 to 1 January 2025.

### 2.2. Participants and Sampling

Purposive sampling was selected to reach the narrowly defined population of licensed orthopedic surgeons practicing in Saudi Arabia who had recent arthroscopic Bankart-repair experience. Eligible surgeons had performed at least five Bankart repairs during the preceding year, worked in an institution providing orthopedic surgery, and provided consent. Surgeons practicing outside Saudi Arabia, non-orthopedic specialists, retired or non-practicing surgeons, and surgeons performing fewer than five Bankart repairs annually were excluded.

Potential participants were approached through professional orthopedic associations and email networks across the five regions. A complete national sampling frame was not available in the study records. The number of surgeons invited, incomplete responses, reminder contacts, and the invitation denominator were not retained; consequently, a response rate and formal assessment of non-response bias could not be calculated. The final descriptive sample comprised 71 eligible respondents.

No a priori sample-size calculation or statistical power analysis was performed. Accordingly, no target population size, expected proportion, or prespecified margin of error informed recruitment; all eligible responses obtained during the study period were analyzed, and the resulting precision was addressed through confidence intervals and explicit limitation statements.

### 2.3. Questionnaire Development and Data Collection

**Survey administration:** The structured electronic questionnaire was distributed through email networks and professional orthopedic associations after obtaining informed consent. Self-administration avoided interviewer prompting; however, the study record did not document whether the survey was anonymous, whether unique links, account restrictions, IP/cookie controls, or manual screening were used to prevent duplicate participation, or whether reminders were sent. No formal social-desirability measure was applied.

**Questionnaire development and adaptation:** The questionnaire comprised demographic/practice characteristics, general knowledge and perceptions, anchor-use preferences, and reported considerations. Anchor-number domains were informed by prior Bankart-repair reports [6,8,9,10,11], anchor-type content by the suture-anchor literature [7], and positioning content by references [13,14]. The literature informed the domains rather than supplying a verbatim validated questionnaire. A source-to-item adaptation matrix, original version history, and documentation distinguishing directly used from modified wording were not retained. No formal translation/back-translation or cross-cultural adaptation was documented; relevance to Saudi practice was assessed by local experts. The final analytical items and response options are provided in Supplementary Table S1.

**Pilot testing and content validity:** The questionnaire was pre-tested for clarity with 20 surgeons. The study record did not establish whether these surgeons were excluded from or overlapped with the 71 main-study respondents; potential overlap therefore could not be ruled out. Three experts rated item relevance on a four-point scale, producing an S-CVI/Ave of 0.911. Item-level I-CVI ratings and the item-level revision/removal log were not retained and could not be reconstructed. Because only three experts participated, the content-validity evidence was interpreted as preliminary and sensitive to each expert’s ratings.

**Reliability:** Demographic variables, knowledge items, practice preferences, and anchor-use questions represented heterogeneous stand-alone constructs and were not summed into a unidimensional scale. Although a preliminary overall Cronbach’s alpha of 0.86 had been calculated, the contributing item set or coherent subscale was not documented; consequently, the coefficient was not interpreted as evidence of the questionnaire-wide internal consistency, and no subscale-specific alpha was reported [12].

**Ethics:** Ethical approval was obtained from the Institutional Review Board of the Regional Research Ethics Committee, Qassim Province, Saudi Arabia (IRB number: 607/46/5792).

### 2.4. Statistical Analysis

Descriptive analyses were conducted in SPSS version 26 for all 71 respondents and were presented as frequencies and percentages; a Wilson 95% confidence interval was calculated for the main proportion. Table 1 percentages used all respondents (n = 71) as the denominator. For the multiple-response anchor-type and selection-consideration items, each percentage represented the number selecting that option divided by 71, so totals could exceed 100%. In Table 2 and Table 3, percentages were calculated within each anchor-preference column.

The original response options were 1–2 anchors, 3–4 anchors, and ‘Both.’ Because ‘Both’ denoted case-dependent use of either count category rather than a single count level, its three respondents were summarized descriptively and excluded from inferential testing. Exploratory associations therefore compared the mutually exclusive 1–2-anchor (n = 18) and 3–4-anchor (n = 50) groups (inferential n = 68). Anchor-type selections were evaluated as separate binary selected/not-selected variables.

Two-sided Fisher exact or Fisher–Freeman–Halton exact tests were used because of sparse cells. Cramér’s V quantified association magnitude. Normal-approximation bootstrap 95% confidence intervals for Cramér’s V were obtained from 10,000 multinomial resamples and truncated at zero where required. Exact *p*-values governed statistical inference, whereas effect sizes and their intervals were interpreted descriptively; Pearson chi-square statistics were not reported in figure legends. Statistical significance was defined as *p* < 0.05.

Alternative binary outcome coding was evaluated as above. Multivariable modeling was not performed because the smaller outcome group contained only 18 participants and several predictor strata remained sparse, which precluded stable adjustment across the candidate variables. All association estimates were therefore explicitly treated as unadjusted and exploratory rather than independent effects. Complete data were available for the 71 respondents; no imputation was performed.

## 3. Results

Among 71 surgeons, 33 (46.5%) practiced in the Central region, 26 (36.6%) had <5 years of orthopedic experience, and 55 (77.5%) had specialized shoulder-surgery training. Thirty-one (43.7%) performed <10 Bankart repairs annually, whereas 11 (15.5%) performed >30. Beach-chair positioning was reported by 49 (69.0%). Anchor type was a multiple-response item: 32 (45.1%) selected suture anchors, 28 (39.4%) selected knotless anchors, and 26 (36.6%) selected all-suture anchors. Fifty surgeons (70.4%; 95% CI: 59.0–79.8%) reported usually using 3–4 anchors, 18 (25.4%) reported 1–2 anchors, and three (4.2%) selected case-dependent ‘Both.’ Thirty-six (50.7%) reported a standard anchor protocol, 20 (28.2%) reported anchor-number-related complications, 55 (77.5%) perceived anchor number to affect surgical success, 46 (64.8%) would retain the same number if cost and other factors were not considered, and 49 (69.0%) considered the current guidance adequate.

The most frequently reported considerations when selecting anchor number were tear size and extent (95.8%), perceived repair quality (94.4%), recurrence reduction (74.6%), surgeon preference (67.6%), and bone quality (66.2%) (Figure 1). These frequencies were descriptive and were not tests of whether the considerations determined anchor-number preference.

In binary exploratory analyses excluding the three case-dependent responses, anchor-number preference was not associated with Saudi region (exact *p* = 0.097; Cramér’s V = 0.335, 95% CI: 0.153–0.516) or years of orthopedic experience (exact *p* = 0.221; Cramér’s V = 0.270, 95% CI: 0.077–0.463) (Table 2).

Anchor-number preference was associated with annual Bankart-repair volume (exact *p* = 0.021; Cramér’s V = 0.371, 95% CI: 0.194–0.548) and hypothetical future preference when cost and other factors were not considered (exact *p* = 0.044; Cramér’s V = 0.298, 95% CI: 0.068–0.529) (Table 3 and Figure 2). Patient positioning was not associated with preference (exact *p* = 1.000; Cramér’s V = 0.022, 95% CI: 0.000–0.167) and was retained in Table 3 without a separate repetitive figure. No other exact association reached statistical significance. All analyses were unadjusted and exploratory.

## 4. Discussion

To our knowledge, this was the first survey describing anchor-number preferences among Saudi orthopedic surgeons performing arthroscopic Bankart repair for anterior shoulder instability. Approximately three-quarters of respondents had received specialized shoulder-surgery training (77.5%), and about seven in ten reported usually using 3–4 anchors (70.4%). Tear size/extent (95.8%) and perceived repair quality (94.4%) were the most frequently reported considerations, followed by recurrence reduction (74.6%), surgeon preference (67.6%), and bone quality (66.2%). These findings described reported practices and perceptions and did not constitute comparative-effectiveness evidence.

The predominance of 3–4-anchor preference was broadly consistent with patterns described in the international literature. However, the survey did not standardize lesion extent, bone loss, capsular laxity, patient risk, anchor configuration, surgical technique, recurrence assessment, or postoperative stability. These omissions precluded conclusions regarding an optimal anchor number or clinical superiority of one preference category.

Some biomechanical and clinical studies reported improved fixation or reduced recurrence with three or more anchors in selected settings [9,10,11], whereas others reported satisfactory outcomes with fewer anchors under different circumstances [8]. Such variation may reflect tear morphology, bone loss, patient characteristics, technique, and follow-up duration. In the present survey, the 77.5% who perceived anchor number to affect success expressed an opinion; no clinical outcomes were measured to verify that perception [10,15].

Beach-chair positioning was reported by 69.0% of respondents and lateral decubitus alone by 22.5%. Both positions have recognized procedural advantages [13,14]. After excluding the three case-dependent anchor responses from inference, positioning was not associated with anchor-number preference (exact *p* = 1.000). The data did not test whether training or personal preference explained positioning; these remained possible explanations only and could not be stated as drivers.

Approximately half of all respondents (50.7%) reported a standard protocol, and 28.2% reported observing anchor-number-associated complications. As those outcomes are self-reported and we have reviewed no medical records, they cannot be considered a complication rate or proof that the protocol improves outcomes. Instead, prior research supports balancing between fixation requirement and hardware burden and preservation of glenoid bone [16,17,18,19].

Tear size/extent was the most frequently reported selection consideration, supporting only the descriptive observation that surgeons commonly considered lesion characteristics. The association between current anchor-number preference and a hypothetical future choice when cost and other factors were removed (exact *p* = 0.044) did not demonstrate that financial factors influenced actual clinical practice, nor did it identify the direction or independence of any cost effect [20,21,22,23,24,25].

In the unadjusted binary analyses, annual Bankart-repair volume (exact *p* = 0.021; Cramér’s V = 0.371, 95% CI: 0.194–0.548) and hypothetical future preference without cost or other constraints (exact *p* = 0.044; Cramér’s V = 0.298, 95% CI: 0.068–0.529) were associated with anchor-number preference. Region, experience, specialized training, positioning, anchor type, protocol use, reported complications, perceived success, and perceived guideline adequacy were not associated under exact testing. Because no multivariable model was fitted, the two observed associations could not be interpreted as independent factors. The hypothesis that 3–4 anchors would be most frequently reported was supported, whereas the hypothesis of variation across practice characteristics was only partially supported.

This study had several limitations. No a priori sample-size calculation or power analysis was performed. Purposive recruitment, the absence of a complete sampling frame, and unavailable invitation and reminder counts prevented calculation of a response rate and formal evaluation of non-response bias. Survey anonymity, duplicate-prevention settings, and other response-control procedures were not documented, and self-reporting remained susceptible to recall and social-desirability bias. Whether the 20 pilot participants overlapped with the 71 main-study respondents could not be determined. Content validity relied on only three experts; item-level I-CVI values and the item-revision log were unavailable. The heterogeneous questionnaire did not support interpretation of a single global Cronbach’s alpha, and no coherent subscale-specific coefficient could be reconstructed. The three case-dependent ‘Both’ responses were too few and conceptually distinct for inclusion as a third inferential outcome category, while the smaller binary group contained only 18 surgeons. Sparse strata and the lack of multivariable adjustment limited precision and precluded independent-factor inference; the effect-size intervals were correspondingly broad. Finally, lesion characteristics, operative technique, and clinical outcomes were not standardized or collected, so causation, cost effects on actual practice, shoulder stability, recurrence, and comparative effectiveness could not be inferred. Strengths included recruitment across five Saudi regions, representation of diverse practice settings, exact testing, explicit effect-size uncertainty, and transparent separation of descriptive and inferential categories.

## 5. Conclusions

Most surveyed orthopedic surgeons in Saudi Arabia reported preferring 3–4 anchors for arthroscopic Bankart repair, and tear size/extent was the most frequently reported selection consideration. In unadjusted exploratory analyses comparing the mutually exclusive 1–2- and 3–4-anchor groups, anchor-number preference was associated with annual surgical volume and with hypothetical future preference when cost and other factors were not considered. These associations could not be interpreted as independent determinants or as evidence that cost influenced actual practice. The survey did not establish superior stability, clinical effectiveness, or a basis for guideline recommendations.

## Figures and Tables

**Figure 1:**
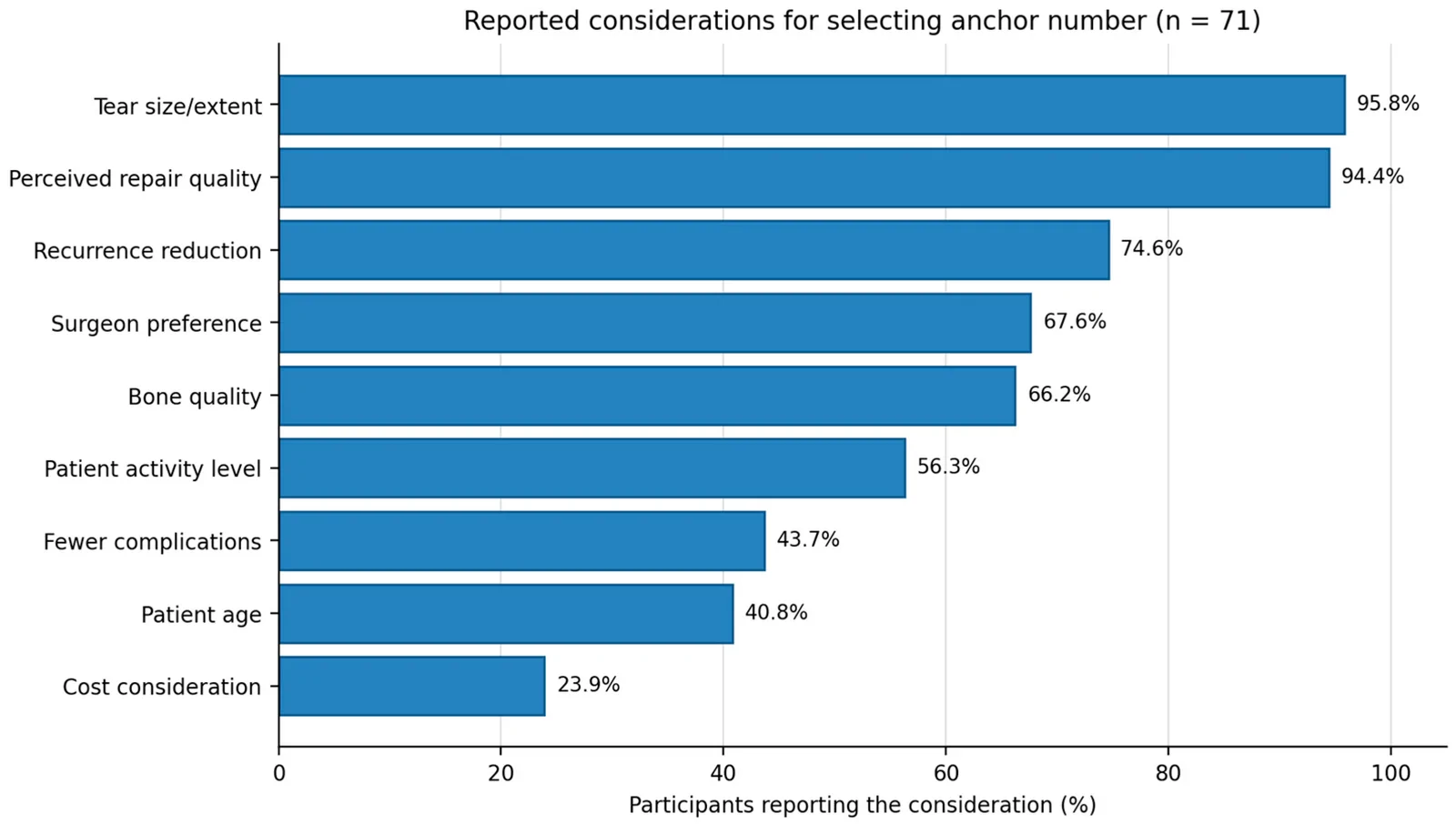
Reported considerations when selecting the number of anchors (n = 71). Multiple responses were allowed; percentages were descriptive and did not represent tested determinants.

**Figure 2:**
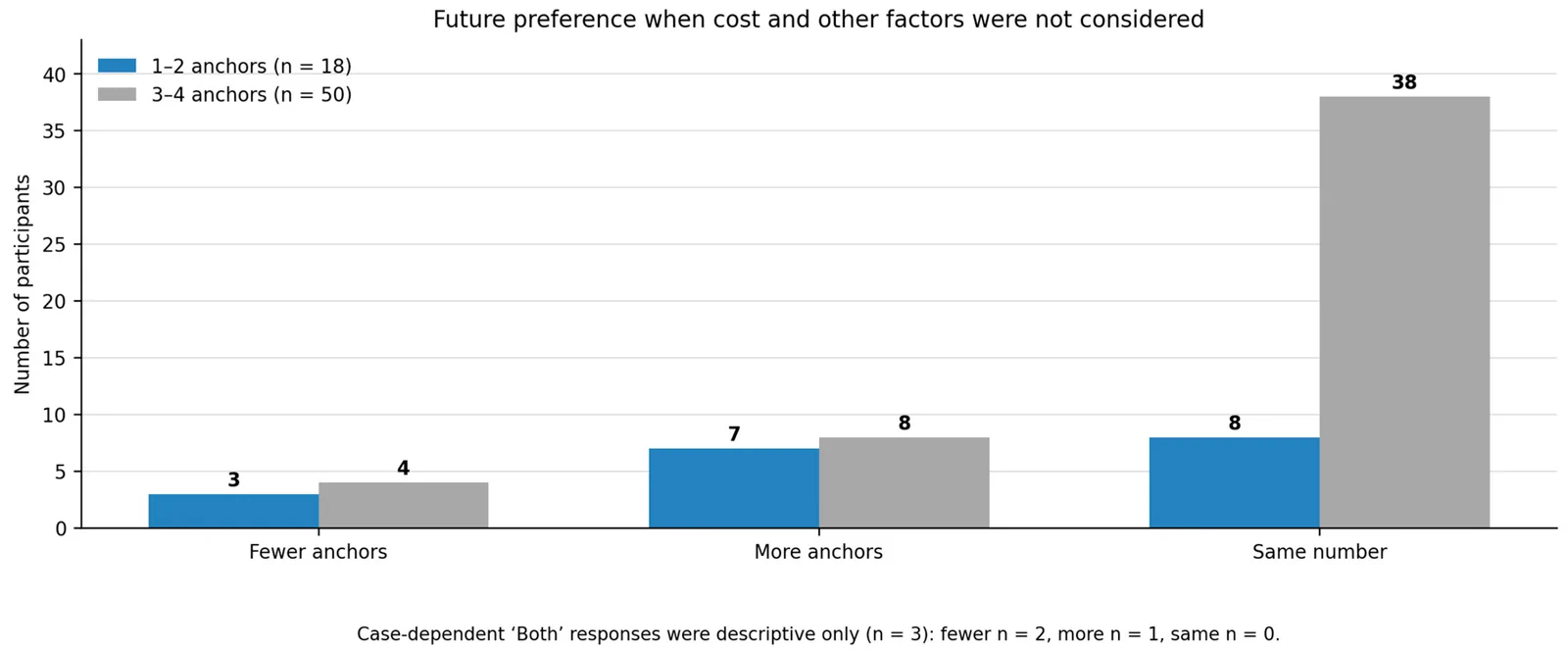
Absolute participant counts for hypothetical future anchor preference among the mutually exclusive 1–2- and 3–4-anchor groups (exact *p* = 0.044; Cramér’s V = 0.298, 95% CI: 0.068–0.529). The three case-dependent ‘Both’ responses were not included in inferential testing and are stated descriptively beneath the graph.

**Table 1:** Participant demographic and work-related characteristics and reported anchor practices (n = 71). Percentages for single-response items used n = 71 as the denominator. Anchor type was a multiple-response item; each percentage used n = 71, and totals could exceed 100%.

Variable	No. (%)
**Region within the Kingdom of Saudi Arabia**	
Central region	33 (46.5)
Eastern region	7 (9.9)
Northern region	1 (1.4)
Southern region	10 (14.1)
Western region	20 (28.2)
**Years of experience as an orthopedic surgeon**	
<5	26 (36.6)
5–10	16 (22.5)
11–20	25 (35.2)
>20	4 (5.6)
**Have you received any specialized training in shoulder surgery?**	
No	16 (22.5)
Yes	55 (77.5)
**On average, how many Bankart repairs do you perform per year?**	
<10	31 (43.7)
10–20	15 (21.1)
21–30	14 (19.7)
>30	11 (15.5)
**What patient positioning do you typically use for Bankart repair?**	
Beach chair	49 (69.0)
Lateral decubitus	16 (22.5)
Both	6 (8.5)
**What type of anchors do you typically use for Bankart repair? (multiple responses allowed)**	
All suture anchors	26 (36.6)
Knotless anchors	28 (39.4)
Suture anchors	32 (45.1)
**On average, how many anchors do you use in a typical Bankart repair surgery?**	
1–2 anchors	18 (25.4)
3–4 anchors	50 (70.4)
Both	3 (4.2)
**Do you have a standard protocol for the number of anchors used in Bankart repair?**	
No	35 (49.3)
Yes	36 (50.7)
**Have you observed any complications related to the number of anchors used?**	
No	51 (71.8)
Yes	20 (28.2)
**In your experience, does the number of anchors used impact the overall success of the surgery?**	
No	16 (22.5)
Yes	55 (77.5)
**Would you prefer to use more or fewer anchors in future surgeries if cost and other factors were not a consideration?**	
Fewer anchors	9 (12.7)
More anchors	16 (22.5)
The same	46 (64.8)
**Do you believe that the current guidelines on anchor usage in Bankart repair are adequate?**	
No	22 (31.0)
Yes	49 (69.0)

**Table 2:** Unadjusted relationship of anchor-number preference with Saudi region and orthopedic experience. Percentages were calculated within each preference column. Case-dependent ‘Both’ responses were descriptive only; exact *p*-values, Cramér’s V, and 95% CIs compared 1–2 versus 3–4 anchors (n = 68).

Variable	On Average, How Many Anchors Do You Use in a Typical Bankart Repair Surgery?			Cramér’s V (95% CI)	Exact *p*-Value
	1–2 Anchors n = 18 No. (%)	3–4 Anchors n = 50 No. (%)	Case-Dependent ‘Both’ n = 3 No. (%) Descriptive Only		
**Region within the Kingdom of Saudi Arabia**					
Central region	11 (61.1)	20 (40.0)	2 (66.7)	0.335 (0.153–0.516)	0.097
Eastern region	1 (5.6)	6 (12.0)	0 (0.0)		
Northern region	1 (5.6)	0 (0.0)	0 (0.0)		
Southern region	3 (16.7)	6 (12.0)	1 (33.3)		
Western region	2 (11.1)	18 (36.0)	0 (0.0)		
**Years of experience as an orthopedic surgeon**					
<5	10 (55.6)	14 (28.0)	2 (66.7)	0.270 (0.077–0.463)	0.221
5–10	3 (16.7)	13 (26.0)	0 (0.0)		
11–20	5 (27.8)	20 (40.0)	0 (0.0)		
>20	0 (0.0)	3 (6.0)	1 (33.3)		

**Table 3:** Unadjusted relationship of anchor-number preference with practice characteristics. Percentages were calculated within each preference column. Case-dependent ‘Both’ responses were descriptive only and excluded from exact tests. Anchor-type selections were multiple-response items analyzed separately as selected versus not selected. Exact *p*-values, Cramér’s V, and 95% CIs compared 1–2 versus 3–4 anchors (n = 68).

Variable	On Average, How Many Anchors Do You Use in a Typical Bankart Repair Surgery?			Cramér’s V (95% CI)	Exact *p*-Value
	1–2 Anchors n = 18 No. (%)	3–4 Anchors n = 50 No. (%)	Case-Dependent ‘Both’ n = 3 No. (%) Descriptive Only		
**Have you received any specialized training in shoulder surgery?**					
No	7 (38.9)	8 (16.0)	1 (33.3)	0.244 (0.000–0.489)	0.093
Yes	11 (61.1)	42 (84.0)	2 (66.7)		
**On average, how many Bankart repairs do you perform per year?**					
<10	11 (61.1)	18 (36.0)	2 (66.7)	0.371 (0.194–0.548)	**0.021**
10–20	1 (5.6)	14 (28.0)	0 (0.0)		
21–30	1 (5.6)	12 (24.0)	1 (33.3)		
>30	5 (27.8)	6 (12.0)	0 (0.0)		
**What patient positioning do you typically use for Bankart repair?**					
Beach chair	13 (72.2)	35 (70.0)	1 (33.3)	0.022 (0.000–0.167)	1.000
Lateral decubitus	4 (22.2)	12 (24.0)	0 (0.0)		
Both	1 (5.6)	3 (6.0)	2 (66.7)		
**What type of anchors do you typically use for Bankart repair?**					
All suture anchors	6 (33.3)	18 (36.0)	2 (66.7)	0.025 (0.000–0.169)	1.000
Knotless anchors	5 (27.8)	21 (42.0)	2 (66.7)	0.129 (0.000–0.315)	0.399
Suture anchors	8 (44.4)	21 (42.0)	3 (100.0)	0.022 (0.000–0.167)	1.000
**Do you have a standard protocol for the number of anchors used in Bankart repair?**					
No	12 (66.7)	22 (44.0)	1 (33.3)	0.200 (0.000–0.413)	0.168
Yes	6 (33.3)	28 (56.0)	2 (66.7)		
**Have you observed any complications related to the number of anchors used?**					
No	14 (77.8)	36 (72.0)	1 (33.3)	0.058 (0.000–0.205)	0.761
Yes	4 (22.2)	14 (28.0)	2 (66.7)		
**In your experience, does the number of anchors used impact the overall success of the surgery?**					
No	7 (38.9)	9 (18.0)	0 (0.0)	0.217 (0.000–0.456)	0.105
Yes	11 (61.1)	41 (82.0)	3 (100.0)		
**Would you prefer to use more or fewer anchors in future surgeries if cost and other factors were not a consideration?**					
Fewer anchors	3 (16.7)	4 (8.0)	2 (66.7)	0.298 (0.068–0.529)	**0.044**
More anchors	7 (38.9)	8 (16.0)	1 (33.3)		
The same	8 (44.4)	38 (76.0)	0 (0.0)		
**Do you believe that the current guidelines on anchor usage in Bankart repair are adequate?**					
No	8 (44.4)	14 (28.0)	0 (0.0)	0.155 (0.000–0.366)	0.245
Yes	10 (55.6)	36 (72.0)	3 (100.0)		

Bold—*p*-value less than 0.05 (significant).

## Data Availability

The original contributions presented in this study are included in the article/Supplementary Materials. Further inquiries can be directed to the corresponding author.

## Supplementary Materials

The following supporting information can be downloaded at: https://www.mdpi.com/article/10.3390/healthcare14193304/s1. Table S1. Questionnaire items and response options represented in the final analytical dataset.
