# Supplementary material for: Factors Associated with Anchor-Number Preference Among Orthopedic Surgeons Performing Bankart Repair in Saudi Arabia: A Cross-Sectional Survey
Source: Healthcare (Basel). 2026 Oct 3;14(19):3304. doi: 10.3390/healthcare14193304 (PMC13665102; doi:10.3390/healthcare14193304)
Supplement: Supplementary file 1 [file healthcare-14-03304-s001.zip › healthcare-4434212-supplementary.pdf]

Table S1. Questionnaire items and response options represented in the final analytical dataset.

Section	Item	Question	Response options / response mode
A	1	Region within Saudi Arabia	Central; Eastern; Northern; Southern; Western
A	2	Years of experience as an orthopedic surgeon	<5; 5–10; 11–20; >20 years
A	3	Specialized training in shoulder surgery	Yes; No
A	4	Average number of Bankart repairs performed per year	<10; 10–20; 21–30; >30
B	5	Patient positioning typically used for Bankart repair	Beach chair; lateral decubitus; both
B	6	Anchor types typically used for Bankart repair	All-suture; knotless; suture anchors (multiple responses allowed)
B	7	Average number of anchors used in a typical Bankart repair	1–2; 3–4; case-dependent use of either category (‘Both’)
B	8	Use of a standard protocol for anchor number	Yes; No
B	9	Observed complications related to anchor number	Yes; No
B	10	Perceived effect of anchor number on overall surgical success	Yes; No
B	11	Future preference if cost and other factors were not considered	Fewer; more; same number
B	12	Perceived adequacy of current anchor-usage guidance	Yes; No
C	13	Considerations reported when selecting anchor number	Tear size/extent; repair quality; recurrence reduction; surgeon preference; bone quality; patient activity; complications; patient age; cost (multiple responses allowed)
